# First Human Use of Shockwave L6 Intravascular Lithotripsy Catheter in Severely Calcified Large Vessel Stenoses

**DOI:** 10.1016/j.jscai.2023.100969

**Published:** 2023-05-19

**Authors:** J.D. Corl, Douglas Flynn, Timothy D. Henry, Dean J. Kereiakes

**Affiliations:** The Heart and Vascular Institute and The Carl and Edyth Lindner Center for Research and Education at The Christ Hospital, Cincinnati, Ohio

**Keywords:** calcium modification, peripheral vascular intervention, vascular calcification

## Abstract

**Background:**

Intravascular lithotripsy (IVL) modifies superficial and deep vascular calcium by delivering pulsatile sonic pressure energy that fractures calcium in situ with the consequent enhancement of transmural vessel compliance, limitation of fibroelastic recoil, and optimization of stent implantation. To date, the use of IVL as an adjunct to facilitate stent implantation has been limited by large target vessel size and eccentricity of calcium distribution.

**Methods:**

The Shockwave L6 IVL balloon delivery system includes 6 sonic energy emitters mounted on the shaft of a 30.0-mm long balloon with diameters ranging from 8.0 to 12.0 mm. The balloon nominal pressure is 4 atm. We describe first human use of this novel IVL delivery system to facilitate covered stent implantation in severely calcified stenoses involving the distal abdominal aorta and bilateral iliac arteries.

**Results:**

Full IVL balloon expansion was achieved at low pressures (3 atm), despite the severity of calcification, with subsequent safe and effective covered stent implantation.

**Conclusions:**

The Shockwave L6 balloon seems to expand the application of IVL to the treatment of severely calcified large vessels, such as the abdominal aorta and iliac arteries.

## Highlights

• Vascular calcification complicates procedural and late outcomes of angioplasty.
• Intravascular lithotripsy modifies calcium to facilitate optimal stent implantation.
• This therapy in large arteries/aorta has been limited by intravascular lithotripsy balloon size.
• Shockwave L6 intravascular lithotripsy balloon safely and effectively treated these aortic/iliac artery stenoses.

## Central Illustration

**Figure:**
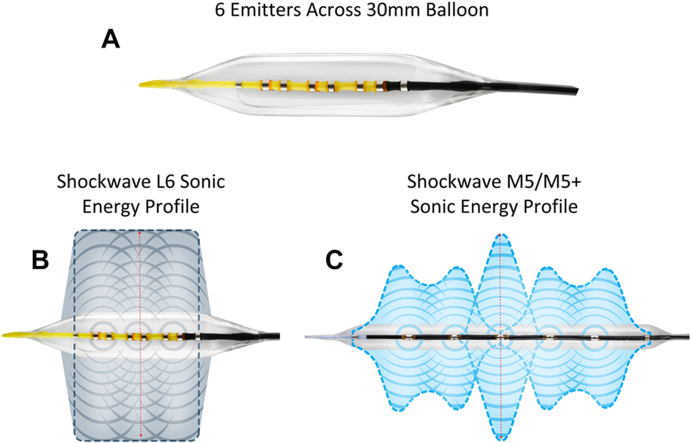

## Introduction

Advanced age and an increasing frequency of diabetes, systemic hypertension, and chronic kidney disease contribute to an increased prevalence and severity of vascular calcification.^1^ Calcified plaque negatively affects procedural, early, and late clinical outcomes after percutaneous vascular intervention. Moderate to severe vascular calcium leads to stent underexpansion, asymmetry, and malapposition,^2^^,^^3^ which may be associated with adverse clinical events such as restenosis and thrombosis.^3^ Multiple technologies have been developed to modify vascular calcification with the intent to optimize stent expansion and improve subsequent clinical outcomes.^4^^,^^5^ These technologies have been limited by target vessel size and the depth and eccentricity of calcium distribution. Intravascular lithotripsy (IVL) modifies both superficial and deep vascular calcium by delivering pulsatile sonic pressure energy that fractures calcium in situ, thus enhancing transmural vessel compliance and limiting fibroelastic recoil with a subsequent optimization of stent expansion.^6^ The safety and the effectiveness of IVL to facilitate optimal coronary stent implantation and to enhance long-term primary vessel patency after femoropopliteal peripheral vascular intervention has been demonstrated in large-scale clinical trials and pooled analyses of trials.^7^, ^8^, ^9^ We report the first human use of a novel, IVL delivery system designed to treat severely calcified aortic and large peripheral artery stenoses.

### Device description

The Shockwave L6 IVL catheter is purpose-built to address severe calcification in large peripheral vessels (Central Illustration A). The balloon catheter is offered in 4 inflated diameters—8.0, 9.0, 10.0, and 12.0 mm—all of which are 30.0 mm in length and feature 6 sonic energy emitters incorporated into the shaft of the balloon. With its compact emitter design, L6 offers a consistent, high sonic energy output across the entire length of the balloon (Central Illustration B), which differs from the energy profile of the M5/M5+ IVL balloon (Central Illustration C). The L6 is a 0.018-inch guide wire compatible to provide support needed in large vessel interventions. The L6 provides low-pressure lesion preparation to minimize complications related to barotrauma, and IVL therapy can be delivered at 2-4 atm with a nominal pressure of 4 atm and rated burst pressure of 6 atm. The L6 balloon provides a maximum of 300 sonic pressure pulses.

**Central Illustration:**
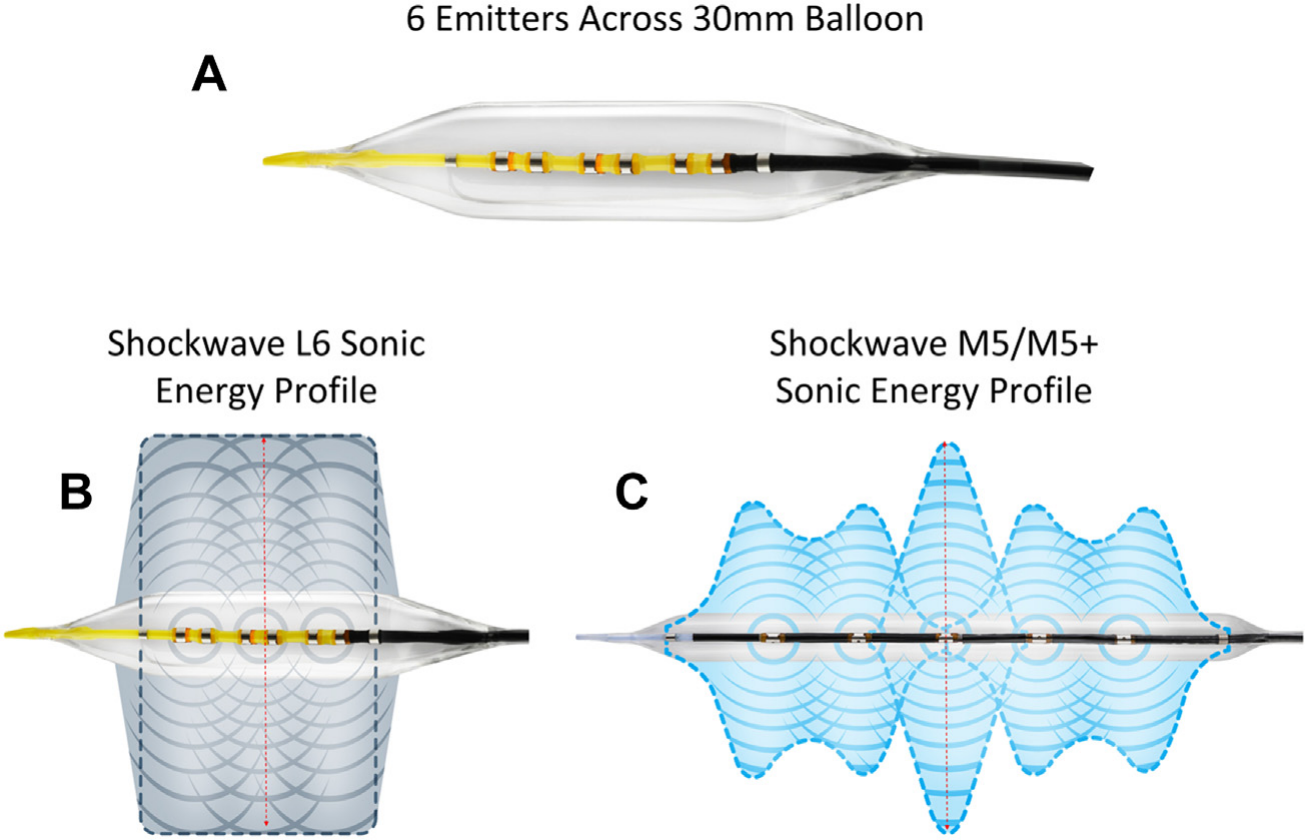
**Shockwave L6 peripheral IVL balloon catheter**. (A) Three channels with 6 emitters along the shaft of the 30 mm balloon. (B) Shockwave L6 sonic energy profile, which is more uniformly intense along the length of the balloon than observed with (C) the Shockwave M5/M5+ peripheral IVL balloon sonic energy profile. The L6 IVL balloon has a unique sonic energy profile. Panel C reproduced with permission from Kereiakes et al.^6^

## Case descriptions

### Aortic case

A 65-year-old woman with a medical history of coronary artery disease, hyperlipidemia, hypertension, and diabetes mellitus was referred for evaluation and treatment of severe, bilateral, lifestyle limiting claudication in August 2022. An aortoiliac duplex study showed a marked velocity elevation (∼400 cm/s) in the distal abdominal aorta consistent with a severe stenosis. Monophasic flow was noted in the bilateral iliac systems.

**Figure 1:**
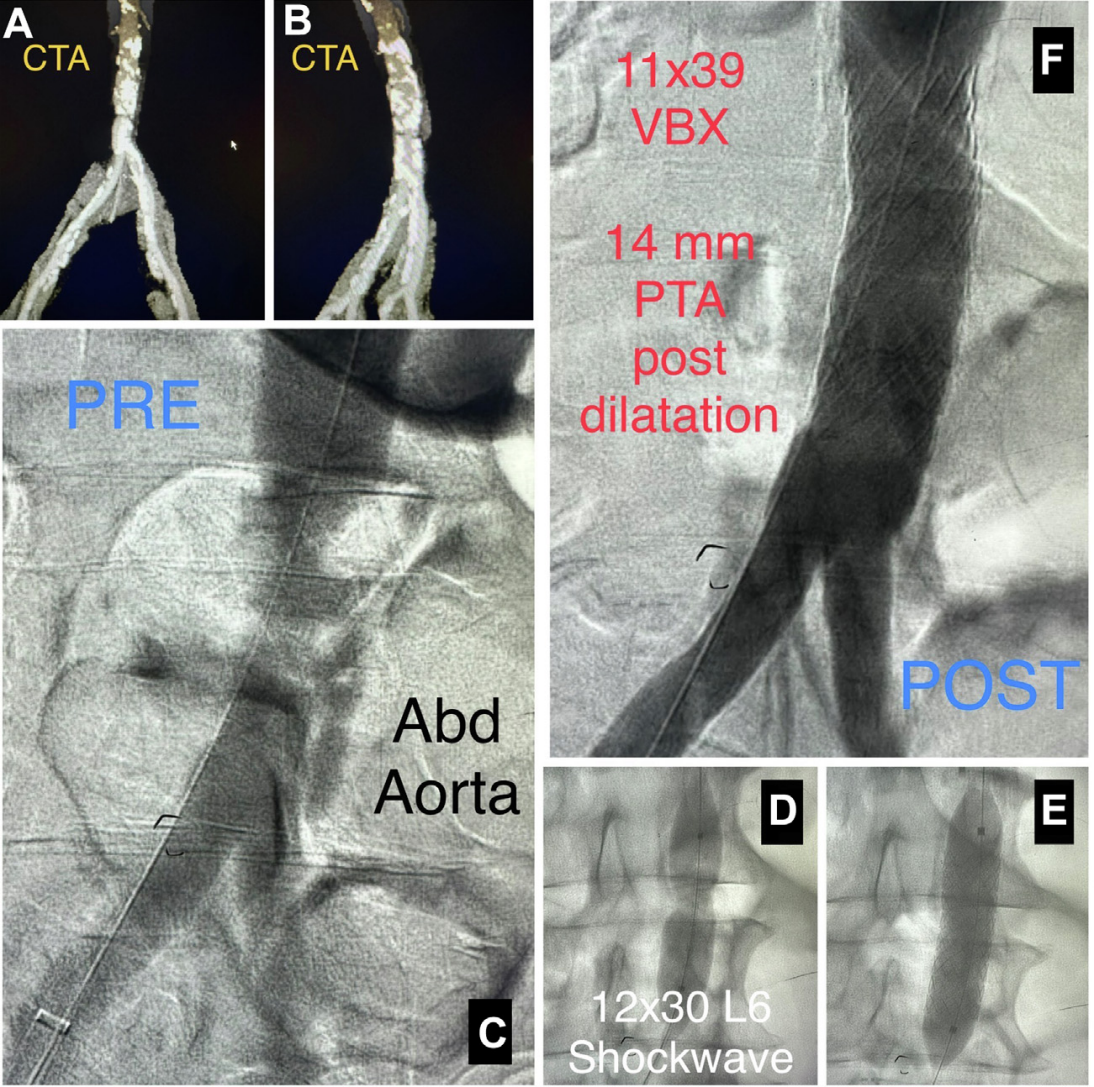
**Abdominal aorta procedural components and imaging.** (A, B) Computed tomography angiography (CTA) of abdominal aorta and iliac arteries. (C) Diagnostic angiogram abdominal aorta. (D) Intravascular lithotripsy (IVL) of abdominal aorta using a 12.0- × 30.0-mm Shockwave L6 balloon. (E) Deployment of 11.0- × 39.0-mm Viabahn VBX balloon expandable covered stent. (F) Final angiogram of abdominal aorta after IVL and stent deployment.

Diagnostic angiography through the right radial artery showed a severely calcified, 90% stenosis of the distal abdominal aorta, 75% stenosis in the distal left superficial femoral artery, and occluded anterior tibial arteries bilaterally. Abdominal computed tomography angiography showed a heavily calcified, greater than 90% stenosis of the infrarenal abdominal aorta with a reference vessel diameter of 12.0-14.0 mm (Figure 1A, B). Revascularization options such as both endovascular and surgical approaches were discussed, and the patient was referred for surgical consultation owing to the severity of vascular calcification. She elected to proceed with an endovascular approach because of the risks associated with surgery.

The patient returned to the catheterization laboratory for the interventional procedure in November 2022. A 25-cm 8F Brite Tip sheath (Cordis) was placed through the right common femoral artery and a 5F sheath was placed through the right radial artery. A 150.0-cm, 0.035-inch NaviCross support catheter (Terumo) was advanced to the abdominal aorta through the right radial sheath for angiographic images during the intervention. An abdominal angiogram was performed (Figure 1C), and a 0.018-inch guide wire was advanced across the abdominal aortic stenosis through the femoral sheath. IVL was performed (180 pulses) in the abdominal aorta using a 12.0- × 30.0-mm Shockwave L6 balloon inflated to 3 atm (Figure 1D). An 11.0- × 39.0-mm Viabahn VBX balloon expandable covered stent (W.L. Gore) was deployed in the abdominal aorta (Figure 1E) and postdilation performed with a 14.0- × 20.0-mm balloon. The final abdominal aortogram showed an excellent angiographic result (Figure 1F).

### Common iliac artery case

A 74-year-old man with a medical history of coronary artery disease, hypertension, diabetes, tobacco use, hyperlipidemia, obstructive sleep apnea, and peripheral artery disease presented with severe bilateral claudication symptoms. Noninvasive vascular study revealed obstructive peripheral artery disease involving the bilateral common iliac arteries (CIAs).

**Figure 2:**
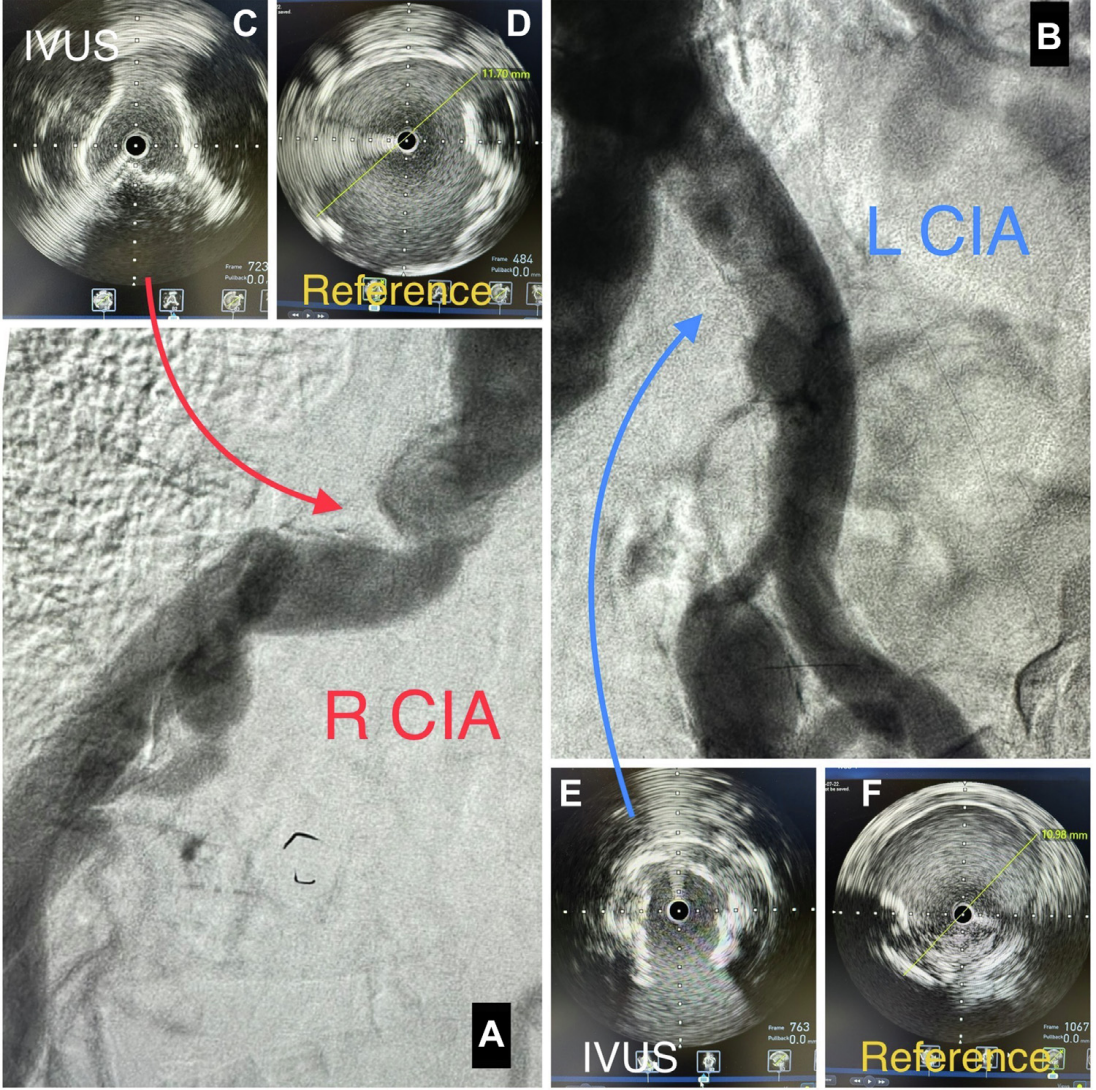
**Diagnostic imaging of bilateral iliac arteries.** (A) Diagnostic angiogram of the right common iliac artery (CIA). (B) Diagnostic angiogram of left CIA. (C) Intravascular ultrasound (IVUS) of severe, heavily calcified stenosis in the right CIA. (D) IVUS of the right CIA reference vessel. (E) IVUS of severe, heavily calcified stenosis in the left CIA. (F) IVUS of the left CIA reference vessel.

Peripheral angiography from the right radial approach revealed severely calcified stenoses involving bilateral CIAs (Figure 2A, B), and the decision was made to proceed with percutaneous revascularization. Bilateral common femoral 8F sheaths were placed using ultrasound guidance.

**Figure 3:**
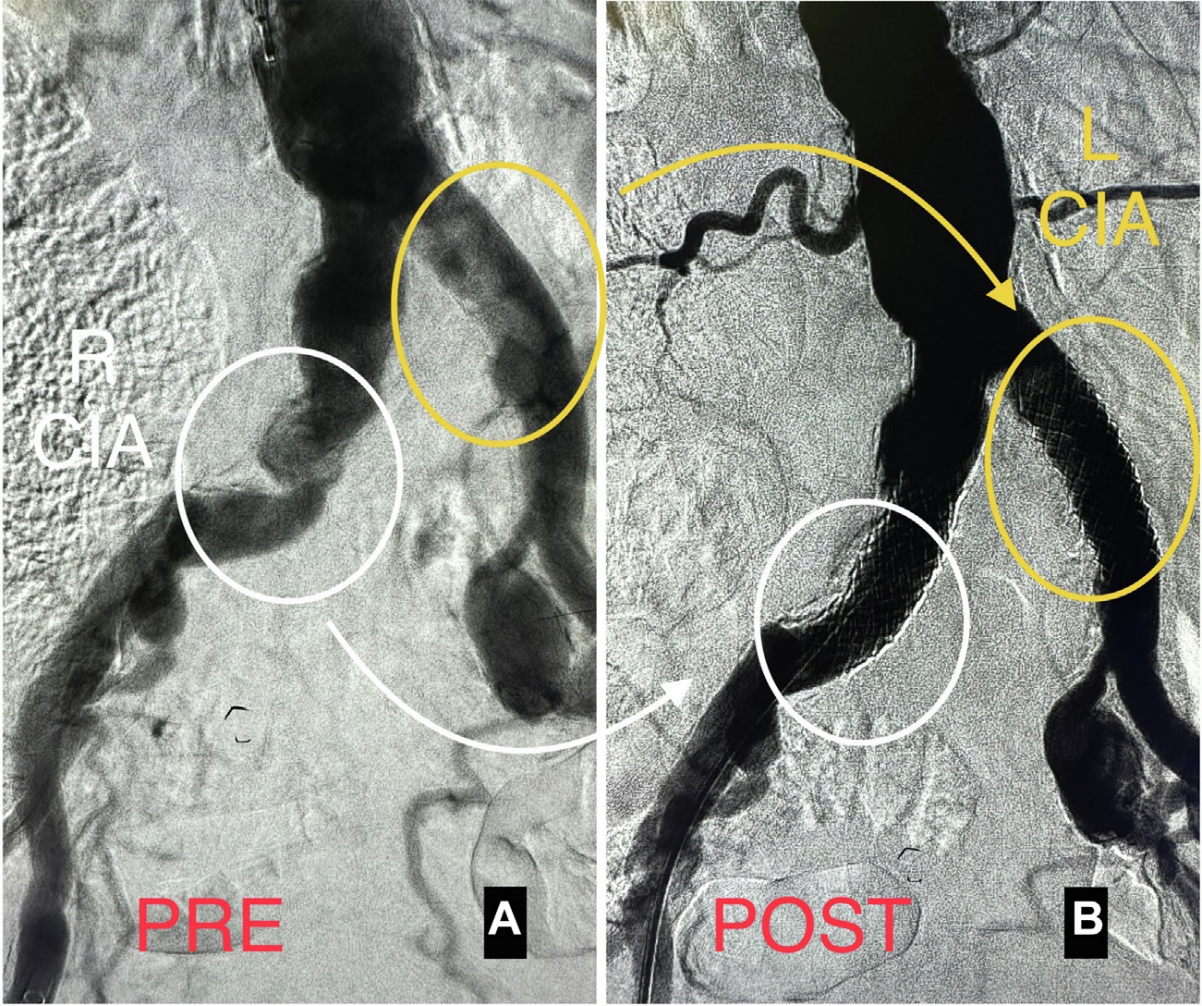
**Preinterventional and postinterventional angiograms.** (A) Preinterventional angiogram of the bilateral iliac arteries. (B) Final angiogram of the bilateral common iliac arteries (CIA) after intravascular lithotripsy (IVL) using a 12.0- × 30.0-mm Shockwave L6 balloon with deployment of an 11.0- × 39.0-mm Viabahn VBX balloon expandable covered stent in each CIA and stent after dilation using a 12.0-mm balloon.

Intravascular ultrasound (IVUS) of right CIA revealed severe calcification with lumen narrowing (Figure 2C) and a reference vessel diameter of 11.7 mm (Figure 2D). The IVUS images of the left CIA also showed a severely calcified stenosis (Figure 2E) and a reference vessel diameter of 10.98 mm (Figure 2F). IVL was performed (150 pulses bilaterally) using a 12.0- × 30.0-mm Shockwave L6 balloon inflated to 3 atm. Viabahn VBX balloon expandable covered stents were deployed bilaterally followed by postdilation with a 12.0-mm diameter balloon in both covered stents. The postinterventional aortogram showed an excellent angiographic result (Figure 3A, B).

## Discussion

Both a severely calcified abdominal aorta stenosis and bilateral CIA stenoses were safely and effectively treated with the novel Shockwave L6 balloon and Viabahn covered stents. Alternative treatment options would have included either surgical grafting/endarterectomy or standard balloon angioplasty, followed by endovascular covered stent deployment. Owing to the density of the calcified plaque in these cases, high-pressure balloon angioplasty would likely have been necessary to achieve effective adequate predilation before stent deployment. Balloon angioplasty in large, heavily calcified vessels carries significant risks including atheroembolization, arterial dissection, and perforation or vessel rupture. These risks are further increased when high pressures are required. Perforation or rupture, specifically involving the abdominal aorta and iliac arteries, may be particularly catastrophic because direct manual compression cannot be applied. IVL can facilitate effective in situ calcium fracture and plaque modification in severely calcified stenoses without the complications associated with barotrauma from high-pressure balloon inflation.^7^, ^8^, ^9^ Indeed, low-pressure (3 atm) L6 balloon inflations achieved full balloon expansion in both cases, and higher balloon pressures were not performed until after covered stent implantation to optimize stent expansion.

In this context, it is important to note that the sonic pressure wave profile of L6 differs from that of the currently available M5/M5+, S4 or C2 IVL catheters.^6^ In the other catheters, the peaks of the sonic pressure waves geographically correlate with the location of the emitters on the shaft of the balloon catheters. The M5/M5+ has the highest sonic pressure wave peak, which corresponds to the middle emitter on the balloon shaft that has its own individual energy source (Central Illustration C).^6^ All other emitters are coupled and share a single energy source between them. Because of the position of the emitters on the shaft of the L6 balloon catheter, the sonic pressure wave peak is higher and more uniform across the surface of the balloon delivery system (Central Illustration B). These initial cases suggest that large vessel (abdominal aorta and iliac artery) calcium modification by the Shockwave L6 IVL balloon is feasible to facilitate stent deployment during percutaneous vascular intervention. Although more clinical experience is required, the Shockwave L6 balloon seems to expand application of IVL to facilitate endovascular intervention in large vessels such as the abdominal aorta and CIAs.
